# Prevalence and predictors of iron deficiency anaemia among children with sickle cell disease in Dodoma, Tanzania: a cross-sectional study

**DOI:** 10.1186/s12889-024-18438-5

**Published:** 2024-04-12

**Authors:** Asha O. Bossy, James J. Yahaya, Shakilu Jumanne

**Affiliations:** 1 Department of Pediatrics and Child Health, School of Medicine and Dentistry, University of Dodoma, Dodoma, Tanzania; 2 Department of Pediatrics and Child Health, Morogoro Regional Referral Hospital, Morogoro, Tanzania; 3 Department of Pathology, School of Health Sciences, Soroti University, Soroti, P. O. Box 211, Uganda

**Keywords:** Sickle cell disease, Iron deficiency anaemia, Predictors

## Abstract

**Background:**

Patients with sickle cell disease (SCD) are prone to iron profile derangements. This study aimed to determine the prevalence of iron deficiency anaemia (IDA) and their predictors among children with SCD aged between 6 months and 14 years. Assessment of the prevalence of IDA and its predictors helps to understand ways of alleviating the magnitude of the problem so as to prevent possible complications such as shortness of breath and chest pain.

**Methods:**

This was a cross-sectional analytical hospital-based study which included 174 patients with SCD attending SCD clinics at St. Gema hospital and Dodoma regional referral hospital in Dodoma city from October 2020 to March 2021. The cut-off points for detection of IDA was serum ferritin level < 30 µg/L and low mean corpuscular volume (MCV) for age. Data were analyzed using SPSS software version 25.0. Multivariate logistic regression analysis was used to determine the predictors of IDA. *P*-value less than 0.05 was considered significant.

**Results:**

The prevalence of IDA in this study was (16.1%, *n* = 28). Family income of less than 70,000/= TZS/month (AOR = 2.2, 95% CI = 1.07–2.49, *p* = 0.023), being transfused with blood less than 3 times from the time of being diagnosed with SCD (AOR = 5.5, 95% CI = 1.03–8.91, *p* = 0.046), and eating red meat at least once per month (AOR = 3.60, 95% CI = 1.37–9.46, *p* = 0.010) remained the independent predictors of IDA in multivariate regression analysis.

**Conclusion:**

The findings of this study have shown that, support of families with children suffering from SCD in terms of financial support for improving medical services including optimal blood transfusion and affordability of diet which is rich in iron such as red meat is imperative.

## Background

Sickle cell disease (SCD) is the world’s most severe and prevalent monogenic hemoglobin disorder [1]. It is characterized by mutation of the β globin gene at position 6 of chromosome number 11 which causes production of rigid β globin chain of a hemoglobin molecule [2]. This leads to less deformable red blood cells (RBCs) during migration process across microvasculature due to formation of sickle shaped RBCs [3]. The hallmark of the pathological process behind major clinical manifestations of SCD are hemolysis and microvascular occlusion [4].

The global burden of SCD is disproportionately high in Africa [5]. Almost 75% of the 300,000 global births of SCD per year occur in Africa and childhood mortality due to SCD remains high ranging between 50% and 90% [6]. SCD occurs at high prevalence in the tropical regions mirroring the geographical distribution of malaria with Nigeria, DR Congo, India and Tanzania as the world’s most prevalent top four countries globally [7]. The incidence of SCD in Tanzania has been reported to be 6 per 1,000 live births corresponding to 11,000 live births annually [8]. A study done in Northwestern, Tanzania reported SCD prevalence of 1.4%, however, the prevalence in Tanzania varies from region to another region [9].

Iron deficiency anaemia (IDA) is highly prevalent in most developing countries where SCD is also very prevalent [10]. Children with SCD in developing countries suffer underlying conditions such as nutritional deficiency and parasitic intestinal infestations which are more likely to cause IDA [11]. A study which was conducted in Northwestern, Tanzania among children aged under-five years reported a prevalence of IDA to be 22.6% [12]. In another study which was done in India, it was found that, 31.1% of the children with SCD had IDA [13]. This high prevalence of IDA has been shown to be higher among children with SCD especially those who do not receive sufficient blood transfusion.

Little is known about the IDA among children with SCD in Tanzania, which underestimates the need for studies in this area to inform the public in general on the formulation of evidence-based guidelines regarding iron supplementation in children with SCD. Therefore, this study aimed to determine the prevalence of IDA and associated factors among children with SCD.

## Methods

### Study design and setting

This was a cross-sectional analytical hospital-based study design, which was conducted at the two hospitals; Dodoma regional referral hospital and St. Gemma hospital in Dodoma city, Tanzania. St. Gema hospital is a faith-based hospital which is at a level of a district hospital whereas Dodoma regional referral hospital is a government hospital and a regional hospital for Dodoma region. At least 18 and 13 children with SCD attend at Dodoma regional referral hospital and St. Gema hospital every month, respectively. Patients attend at the clinics at least once a month, but they may sometimes attend twice per month depending on their condition. Various services are provided during SCD clinic days including comprehensive treatment and management, pain management, patient education, and psychiatric services.

### Patients’ characteristics and recruitment

The inclusion criteria included children with age between 6 months and 14 years who were confirmed using haemoglobin electrophoresis and those with absence of symptoms and/or signs related to acute illness for at least 4 weeks before being enrolled in the study. Other patients with impaired iron absorption or increased body iron deposition like peptic ulcer disease, having malignancy, and children with a history of receiving iron supplements for the past 3 months before commencement of the study were excluded from the analysis.

### Sample size determination and sampling technique

The sample size was calculated using Kish & Leslie’s formula [14]: n = t^2^ x p (1-p)/m^2^; where n = sample size, t = confidence level at 95% (standard value of 1.96), margin of error of 5%, and p = proportion of the estimated prevalence of IDA of 13% from a previous study which was conducted in Dar-es-salaam, Tanzania among children with SCD [15]. A total sample of 174 children with SCD was obtained, and they were voluntarily selected using convenience sampling method where all patients who met the inclusion criteria were included in the analysis.

### Data collection

Data were collected during clinic days using a self-designed data collection form. The research tool was designed by two researchers; SJ (paediatrician) and JJY (pathologist) based on previous literature. Two registered nurses from each hospital were responsible for data collection. The research assistants were nurses that have experience of working in the SCD units in the respective hospitals. The research assistants were first trained on data collection by the researchers prior data collection. Information regarding sociodemographic characteristics, feeding behavior, clinical characteristics, and laboratory investigations were recorded. For children who could not provide the required information, parents and/or guardians were required to provide the information after they had consented on behalf of their children. Blood samples were collected on the day of interview, however, containers for stool samples were given to the parents and/or guardians for the children to collect the stool sample and the samples were submitted during subsequent clinics. Physical examination was performed to examine for signs of IDA such as pallor and vital signs including temperature, pulse rate, respiration rate, and saturation were taken together with anthropometric measurements.

### Detection of hookworm infection

The methodology of testing stool for occult blood used in the present study was adapted from the study which was done previously [16].

### Testing for microhaematuria

Testing of the presence of microhaematurria was based on the procedures as it was done in a previous study [17].

### Blood sample collection and measurement of iron deficiency

Under aseptic technique, approximately 3 mls of venous blood sample was collected from all the patients. Venipunctures containing ethylene diaminetetra acetic acid (EDTA) with purple and red caps were used to collect the blood sample for full blood picture (FBP), serum ferritin (SF), and C-reactive protein (CRP) testing. From FBP, three blood indices were obtained including haemoglobin (Hb) level, mean corpuscular volume (MCV), and mean corpuscular haemoglobin (MCH). FBP was measured using CELL-DYN Ruby hematology (Genway Biotech, USA). SF was used to measure the iron status quantitatively according to the WHO report of 2020 (WHO, 2020) [18]. The cut-off points for detection of IDA was SF level of < 30 µg/L and low MCV for age [19]. SF was measured using Snibe Maglumi 800 analyzer (Genway Biotech, USA).

### Data analysis

Data were analyzed using SPSS software version 25.0. Categorical variables were summarized in frequency and percentages whereas continuous variables were summarized in mean ± standard deviation (SD). Binary logistic regression analysis was used to determine the predictors of IDA. All variables which were statistically significant in univariate analysis were further carried into multivariate analysis to determine the predictors after controlling for confounders. *P* < 0.0.5 was considered significant.

## Results

### Sociodemographic and dietary intake characteristics of the study participants

The sociodemographic and dietary intake characteristics of the patients are presented in Table 1. The mean ± SD age of the patients was 33.5 ± 6.8 months with age range between 6 months and 14 years. Most of the children (52.3%, *n* = 91) were males and also over half (56.9%, *n* = 99) of the children had more than 5 years. The vast majority (81.6%, *n* = 142) of the children were reported to have a habit of drinking cow’s milk. Of all the subjects, only (4.6%, *n* = 8) had access to eat red meat frequently.

**Table 1:** Sociodemographic characteristics and dietary intake of the children (*N* = 174)

Variable	Frequency (n)	Percentage (%)
**Age (months)**		
<24	25	14.4
24–60	50	28.7
>60	99	56.9
**Sex**		
Males	91	52.3
Females	83	47.7
**Drinking cow’s milk**		
Yes	142	81.6
No	32	18.4
**Eating of green vegetables**		
Frequently	57	32.8
Weekly	110	63.2
Monthly	7	4.0
**Eating of fruits**		
Frequently	51	29.3
Weekly	118	67.8
Monthly	5	2.9
**Eating red meat**		
Frequently	8	4.6
Weekly	120	69.0
Monthly	46	26.4

### Sociodemographic characteristics of the caregivers

The sociodemographic characteristics of the caregivers are presented in Table 2. The mean ± SD age of the caregivers was 33.5 ± 6.8 years and majority of them (74.1%, *n* = 129) were females. Most of the caregivers (42.0%, *n* = 73) were between 37 and 47 years of age. Also, most of the caregivers (48.3%, *n* = 84) had attained secondary education, and over half of the caregivers (58.0%, *n* = 101) were residing in urban area. In addition, over half (58.0%, *n* = 101) of the caregivers had a family income ranging between 70,000 and 300,000/= TShs.

**Table 2:** Sociodemographic characteristics of caregivers (*N* = 174)

Variable	Frequency (n)	Percentage (%)
**Age (years)**		
≤25	27	15.5
26–36	72	41.4
37–47	73	42.0
>47	2	1.1
**Sex**		
Male	45	25.9
Female	129	74.1
**Education level**		
Informal education	8	4.6
Primary education	46	26.4
Secondary education	84	48.3
Tertiary education	36	20.7
**Marital status**		
Married/cohabiting	89	51.1
Single	38	21.8
Separated/divorced	28	16.1
Widow/widower	19	10.9
**Place of residence**		
Rural	73	42.0
Urban	101	58.0
**Occupation**		
Employed	38	21.8
Self employed	38	21.8
Unemployed	98	56.4
**Family income/month (TShs)**		
<70,000.00	37	22.5
70,000-300,000.00	101	58.0
>300,000.00	34	19.5

### Clinical characteristics among study subjects

The clinical characteristic and laboratory investigations are presented in Table 3. The vast majority (68.5%, *n* = 121) of the children had ≥ 2 years at the age of diagnosis of SCD with mean ± SD age at diagnosis of SCD 3.0 ± 1.0 years. Also, majority (89.1%, *n* = 155) of the subjects were reported to have had experienced SCD crises. Hydroxyurea use among children was reported in almost one-third (27.0%, *n* = 47) of all the children.

**Table 3:** Clinical characteristic of the children (*N* = 174)

Variables	Frequency (n)	Percentage (%)	Mean ± SD
**3 months history of blood transfusion prior enrollment in the study**			
Yes	137	78.7	
No	37	21.3	
**Frequency of blood transfusion**			2.2 ± 1.1
<3 times	167	96.0	
≥3 times	7	4.0	
**History of hydroxyurea use**			
Yes	47	27.0	
No	127	73.0	
**Age at SCD clinic attendance**			2.3 ± 1.4
<2 years	55	31.6	
≥2 years	119	68.4	
**Age at SCD diagnosis**			3.0 ± 1.0
<2 years	53	30.5	
≥2 years	121	68.5	
**SCD crises in the past 6 months**			
Yes	155	89.1	
No	19	10.9	
**Number of SCD crises in the past 6 months**			1.8 ± 1.0
≤3	149	96.1	
>3	6	3.9	
**Frequency of SCD crises**			
Very often	12	7.7	
Sometimes	143	92.3	
**Jaundice**			
Yes	26	14.9	
No	148	85.1	
**Pallor**			
Yes	74	42.5	
No	100	57.5	
**Chest pain**			
Yes	25	14.4	
No	149	85.6	

### Prevalence of iron deficiency anaemia among study subjects

The results for the various laboratory investigations which were carried out among study subjects are presented in Table 4. IDA (low SF level) was found in (16.1%, *n* = 28) of all the children. Severe anaemia, hookworm infection, and positive occult blood test were found in only (1.2%, *n* = 2), (2.9%, *n* = 5), and (8.0%, *n* = 14) of all the children, respectively.

**Table 4:** Prevalence of IDA among study subjects (*N* = 174)

**Variables**	**Frequency (n)**	**Percentage (%)**	**Mean ± SD**
**Serum ferritin level (µg/L)**			155.7 ± 203.7
Normal	137	78.7	
Low	28	16.1	
High	9	5.2	
**Hb level (gm/dL)**			8.1 ± 1.2
Mild (10.0-10.9)	153	87.9	
Moderate (7.0-9.9)	19	10.9	
Severe (< 7.0)	2	1.2	
**MCV (fL)**			72.9 ± 9.3
Normal (72–115)	168	96.6	
Low (< 72)	5	2.9	
High (> 115)	1	0.5	
**MCH (pg)**			24.0 ± 4.3
Normal (25–40)	168	96.6	
Low (< 25)	5	2.9	
High (> 40)	1	0.5	
**Stool for hookworm ova**			
Positive	5	2.9	
Negative	169	97.1	
**Stool for occult blood**			
Positive	14	8.0	
Negative	160	92.0	
**Urine for RBCs**			
Positive	5	2.9	
Negative	169	97.1	

### Predictors of IDA among children with sickle cell disease

In multivariate logistic regression analysis, children from families with family income below the International Poverty Line (IPL) of 150,000 TZS/month (average 2.0 USD/day) were 2.2 times significantly more likely to have IDA compared with children whose family income was above IPL (95% CI = 1.07–2.49, *p* = 0.023). Children who had a history of getting blood transfusion < 3 times were 5.5 times significantly more likely to be diagnosed with IDA than children who had ≥ 3 times (95% CI = 1.03–8.91, *p* = 0.046). Also, children who were eating red meat not at least weekly, were 3.6 times significantly more likely to develop IDA than children who were eating red meat at least once per week (95% CI = 1.37–9.46, *p* = 0.010) (Table 5).

**Table 5:** Binary logistic regression analysis for determination of the predictors of IDA

	Iron deficiency anaemia		Univariate analysis		Multivariate analysis	
Variables	Yes: n (%)	No: n (%)	COR (95% CI)	p	AOR (95% CI)	p
**Family income for the child**						
< 150,000 TZS/month	12 (18.8)	52 (81.2)	0.3 (0.08–0.91)	0.036	2.2 **(**1.07–2.49)	0.023
150,000-500,000 TZS/month	12 (15.2)	67 (84.8)	1.1 (0.32–3.68)	0.889	1.1 (0.31–3.61)	0.930
> 500,000 TZS/month	4 (12.9)	27 (87.1)	Reference		Reference	
**Sex of the parent/caregiver**						
Male	4 (8.9)	41 (91.1)	2.3 (0.77–7.17)	0.136	-	
Female	24 (18.6)	105 (81.4)	Reference			
**Sex**						
Female	17 (20.5)	66 (79.5)	1.9 (0.82–4.28)	0.101	-	
Male	11 (12.1)	80 (87.9)	Reference			
**Frequency of blood transfusion since diagnosis of SCD**						
< 3 times/year	25 (14.9)	143 (85.1)	5.7 (1.09–9.96)	0.039	5.5 **(**1.03–8.91)	0.046
≥ 3 times/year	3 (1.7)	3 (1.7)	Reference		Reference	
**Frequency of sickle cell crises in the last 6 months**						
Very often	0 (0.0)	13 (100.0)	1.4 (0.43–4.68)	0.568	-	
Rarely	24 (16.8)	119 (83.2)	0.4 (0.60–7.11)	0.999	-	
None	4 (22.2)	14 (77.8)	Reference			
**Eating of red meat**						
At least once per month	15 (31.2)	33 (68.8)	4.8 (1.95–5.86)	0.001	3.6 **(**1.37–9.46)	0.010
At least once per week	12 (10.0)	108 (90.0)	1.4 (0.09-2.00)	0.628	0.4 (0.08–2.22)	0.304
Frequently	1 (12.5)	7 (87.5)	Reference		Reference	
**Jaundice**						
Yes	2 (7.7)	24 (92.3)	0.4 (0.09–1.76)	0.221	-	
No	26 (17.6)	122 (82.4)	Reference			
**Urine for RBCs**						
Positive	2 (40.0)	3 (60.0)	3.7 (0.35–23.03)	0.166	-	
Negative	26 (15.4)	143 (84.6)	Reference			

## Discussion

We aimed to assess the prevalence and associated factors of IDA among children with SCD. The prevalence of IDA in our study was 16.1% and it was associated with reduced frequency of eating red meat, low family income, and less frequency of blood transfusion per month. The prevalence of IDA in the subjects in the present study was close to that of 13.0% which was reported in the study of Mangosongo et al. in Tanzania [15] and 13.3% from the study of Kassim et al. which was done in Yemen [20]. However, lower than the prevalence of 9.6%, 3.1%, 0.0% of IDA in children with SCD that have been reported in India [21], and Nigeria [22] and another study which was also done in Nigeria [23], respectively. The prevalence of 31.0% of IDA which is quite high has also been reported in the study of Patel et al. from Western India [13]. In another study which was done in Jamaican children with SCD, a higher prevalence of 41.7% than the one reported in the present study was reported [24]. The discrepancy in the prevalence of IDA observed in different studies may be due to a number of factors including heterogeneity of the study subjects included in the specific studies, especially when there is no uniformity of the study subjects for the compared studies, is more likely to give variation in the prevalence. Lack of universal consensus regarding cut-off point for identification of IDA cases could also be one of the reasons for the variation in prevalence of IDA. For example, in a study of Patel et al. in which the cut-off of SF was < 50 gm/dL, in one way or another, contributed to the high prevalence of IDA. This is because, having such a cut-off point, is more likely to include even cases without IDA due to fact that, IDA in SCD is not so common. The observed prevalence of IDA among children with SCD in this study show that, still there is a need of considering cases individually in order to prevent underdiagnosis of IDA in patients with SCD particularly in low-and middle-income countries (LMICs) where affordability of good nutrition sometimes is a challenge. Our findings have also shown that, patients with SCD in LMICs where nutrition may be a challenge in some families, therefore, it is important for clinicians to consider each case differently rather than making assumption that patients with SCD for them, IDA is not a problem.

Blood transfusion which involves replacement of sickle RBCs with non-sickle transfused RBCs [25], is of paramount importance in the management of patients with SCD. Blood transfusion helps to prevent both acute and chronic complications of SCD [26]. However, blood transfusion should always be guided with specific reasons for the decision to transfuse or not to transfuse and must always be based on the condition of the patients due to high possibility of complications such as iron overload [25, 27]. In this study, it was found that subjects who had blood transfusion less than 3 times per year had increased odds of developing IDA compared with subjects who had blood transfusion 3 times or more per year. This is in agreement with the finding in the study of Akodu et al. that was done in Nigeria in which it was observed that all subjects with IDA were those who had not received blood transfusion [28]. Also, in another study of Virnchnsky et al. which was done in the United States, it was found that subjects who had not received blood transfusion had increased odds of developing IDA compared with other subjects that were transfused [29].

Dietary intake influences the concentration of iron in serum. In this study, participants who had a history of eating red meat only once per month had increased odds of developing IDA compared to those who were eating red meat at least daily or per week. Similar findings were also reported in the study of Kamal et al. in Saudi Arabia in which two groups (nourished and malnourished patients), and it was found that patients who were eating foods rich in protein had good nutritional status and high mean SF level compared to their counterparts [30]. In another study of Williams et al. it was observed that, poor nutritional status particularly due to lack of protein was associated with poor quality of life and increased risk of developing anaemia [31]. Furthermore, Gibson and Ashwell reported that, eating red meat was associated with good iron status and prevention of IDA [32]. Socioeconomic status (SES) of the family plays a major role in influencing the health of children with SCD through various ways including access to healthy nutrition [33], purchasing of necessary drugs such as hydroxyurea, and medical checkups among many others [34]. In this study, low family income was associated with IDA. This is similar to the finding in the study which was done in Nigeria, in which children with SCD who were living in families with better financial income were had reduced risk of developing anaemia [23]. Furthermore, Olatunya et al. reported from Nigeria that, children with SCD from families with low SES based on low educational level of the head of the household and family income below IPL were associated with severe anaemia and poor nutritional status [35]. This clearly shows that, feeding on balanced diet for patients with SCD is crucial and imperative for the improvement of their health and prevention of anaemia and also prevention of both acute and chronic SCD related complications.

### Strenghts and limitations of the study

The strength of this study is that, the data used to generate the results have been measured consistently and accurately due to forward (prospective) direction of data collection. The study limitations include lack of generalizability of the results due to small sample size. Secondly, the causal-effect relationship cannot be established to cross-sectional nature of the study. Additionally, there was a financial constraint which contributed to failure of including other markers for assessing SF level such as transferritin saturation, transport iron binding capacity (TIBC), erythrocyte protoporphyrin, and transferrin receptor (TfR).

## Conclusion

This study reports quite a significant proportion of children with IDA from a cohort of children with SCD in a limited-resources setting. Also, it was observed that, low family income, being transfused with blood less than 3 times from the time of being diagnosed with SCD, and eating red meat at least once per month were associated with IDA. Therefore, emphasis should be made for clinicians and other stakeholders the importance of evaluating children with SCD for possibility of having IDA so as to prevent them from its complications.

## Data Availability

The datasets analyzed during the current study are not publicly available due to lack of participant consent to share data outside the team of investigators but are available from the corresponding author on reasonable request.
